# Statistical Optimization of Novel Medium to Maximize the Yield of Exopolysaccharide From *Lacticaseibacillus rhamnosus* ZFM216 and Its Immunomodulatory Activity

**DOI:** 10.3389/fnut.2022.924495

**Published:** 2022-06-02

**Authors:** Liang Chen, Qing Gu, Tao Zhou

**Affiliations:** Key Laboratory for Food Microbial Technology of Zhejiang Province, School of Food Science and Biotechnology, Zhejiang Gongshang University, Hangzhou, China

**Keywords:** exopolysaccharide, *Lacticaseibacillus rhamnosus*, statistical optimization, immunomodulatory activity, NF-κB

## Abstract

The traditional media used for the fermentation of *Lactobacilli* always contain carbohydrate polymers, which interfere with the analysis of the exopolysaccharide (EPS) produced by the bacteria. In this investigation, a novel medium formulation that could avoid such interference was successfully developed. The beef extract, yeast extract, and peptone used in this formulation were subjected to the removal of polysaccharides before use. The factors affecting the EPS production were optimized by a single factor test, Plackett–Burman design, and Box–Behnken design. The optimum formula was ascertained as: 7.5 g L^–1^ yeast extract, 12.5 g L^–1^ beef extract, 10 g L^–1^ peptone, 21.23 g L^–1^ maltose, 5.51 g L^–1^ yeast nitrogen base, 2 g L^–1^ K_2_HPO_4_, 5 g L^–1^ anhydrous sodium acetate, 2 g L^–1^ ammonium citrate, 0.58 g L^–1^ MgSO_4_⋅7H_2_O, 0.25 g L^–1^ MnSO_4_⋅H_2_O, and 1 mL L^–1^ Tween 80. The initial pH of the medium was 6.5. The optimized conditions for fermentation of the strain to produce EPS were as follows: seed size 1%, culture temperature 37°C, and culture time 20 h. Optimum results showed that EPS yield was 496.64 ± 3.15 mg L^–1^, being 76.70% higher than that of unoptimized conditions (281.07 ± 5.90 mg L^–1^). The EPS was mainly comprised of glucose and guluronic acid, with a weight average molecular weight of 19.9 kDa; it was also characterized by Fourier transform infrared spectroscopy and UV analysis. EPS was found to significantly enhance the phagocytic capacity, promote the NO, TNF-α, IL-1β, and IL-6 secretion, and improve mRNA expression of cytokines in RAW 264.7 macrophages, indicating its considerable immunomodulatory activity. Western bolt and immunofluorescence results demonstrated that the EPS was able to increase p65 nuclear translocation in the macrophages, indicating that EPS enhanced immunity via the NF-κB signaling pathway. EPS investigated in this work has potential as an attractive functional food supplement candidate for the hypoimmunity population.

## Introduction

The exopolysaccharide (EPS) secreted by *Lactobacilli* organisms, which was generally regarded as safe (GRAS), has been widely used in food-, medicine-, and cosmetics-related industries (1). Recently, studies have demonstrated that *Lactobacilli* EPS possesses remarkable immunostimulatory effects with no obvious side effects (2). EPS maintains the stability of the immune system possibly by regulating the metabolism of the body, such as preventing DNA damage, and controlling blood lipid levels (3); enhancing specific cellular functions, such as improving humoral immune response against antigens by stimulating the proliferation of T/B-lymphocytes, and improving the phagocytic ability of macrophages by promoting their proliferation (4–6).

Culture conditions, such as medium, pH, and temperature are very important factors influencing bacterial growth. Skim milk medium, M17 broth medium, and MRS broth medium (7–9) have been widely used for cultivating *Lactobacilli*. Beef extract, yeast extract, and peptone are often used as organic carbon and nitrogen sources in those media for the fermentation of *Lactobacilli*. A lot of polysaccharide-analogs derived from those components cannot be utilized by the microorganism and would precipitate together with EPS when using the alcohol precipitation method to prepare EPS (10). Thus, the use of these components in the culture medium would interfere with the analysis and quantification of the EPS produced by the bacteria (11). In an attempt to eliminate this interference and facilitate EPS analysis, semi-defined media (12) and chemically defined media (13) have been developed. Although the test strains grew well in these media, in which some components were reduced, substituted, or removed, these media might not be suitable for the growth of other bacterial strains. Hence, eliminating the interference of medium components, screening and optimizing the key parameters that affect the yield of EPS are crucial for the development of a culture method suitable for the EPS production by *Lactobacilli*.

Recently, a bacterial strain was isolated from fresh milk in our laboratory and identified as *Lacticaseibacillus rhamnosus* ZFM216. This bacterium was found to possess a potential for EPS production with a high yield in a preliminary study. The objective of this work is to develop a medium that is suitable for the production of EPS by this strain, and avoid the inference of components in medium to EPS analysis. To this end, several statistical methods, including single factor design, Plackett–Burman design, and Box–Behnken design, were employed to optimize the formulation of the medium and culture conditions. The immunomodulatory activities and action mechanism of EPS obtained under the optimal conditions are also reported.

## Materials and Methods

### Strains and Reagents

*Lacticaseibacillus rhamnosus* ZFM216 was isolated from fresh milk and cultured on MRS agar (14). The strain was identified according to their morphological characteristics and 16S rDNA sequences analysis (data not shown). *L. rhamnosus* ZFM216 has been deposited in the China Center for Type Culture Collection (CCTCC) under accession number NO. CCTCC M 2020325. Macrophage RAW264.7 cells were kindly provided by the College of Pharmaceutical Science, Zhejiang University. Beef extract, yeast extract, and peptone were purchased from Hangzhou Microbial Reagent Co., Ltd. (Hangzhou, China). Glucose, galactose, fructose, sucrose, maltose, lactose, lipopolysaccharide (LPS), and neutral red were obtained from Sigma Chemical Co., Ltd. (St. Louis, MO, United States). Yeast nitrogen base (YNB) was purchased from Shanghai Yuanye Bio-Technology Co., Ltd. (Shanghai, China). The kits for MTT, NO, and total protein assays were purchased from Nanjing Jiancheng Bioengineering Institute (Nanjing, China). ELISA kits for the mouse IL-1β, IL-6, and TNF-α assays were supplied by Lanpai BIO (Shanghai, China). All other chemical reagents used in this study were of analytical grade.

### Monitoring the Change in Polysaccharide Content in the Culture Medium During Fermentation

The strain *L. rhamnosus* ZFM216 was revived twice by incubating on MRS agar at 37°C for 48 h before use. A single colony was inoculated in 10 mL of MRS broth and cultivated at 37°C for 24 h, and then the bacterial suspension (1 mL, 1 × 10^8^ cells mL^–1^) was transferred into an MRS broth (100 mL) for 32-h cultivation at 37°C. Isolation of polysaccharides was carried out according to a published protocol with some modifications (15). After fermentation, the broth (100 mL) was heated to 100°C in a water bath, then the bacteria were removed by centrifugation (10,000 rpm, 15 min). The resulting filtrate was concentrated on a rotary evaporator to about one-fifth of the original volume (about 20 mL), then treated with 800 mg mL^–1^ of TCA (final concentration was 40 mg mL^–1^) for 12 h at 4°C, followed by centrifugation (10,000 rpm, 15 min) to remove protein. A quadruple volume of pre-chilled anhydrous ethanol was then added and allowed to stand for 12 h at 4°C. The precipitate was obtained by centrifugation (10,000 rpm, 15 min), then redissolved in 10 mL of ultrapure water and dialyzed (M_*W*_ cut off 2000 Da) against deionized water for 72 h. The dialyzate was concentrated and then underwent freeze-drying to obtain polysaccharides. The polysaccharide content was monitored by measuring the total sugar content (16) at 2-h intervals during the period of fermentation. In addition, the polysaccharide contents of beef extract, peptone, and yeast extract in the MRS medium were also determined according to the above methods. All the experiments were performed in triplicate.

### Modification of MRS Medium

The traditional MRS was composed of 5 g L^–1^ yeast extract, 10 g L^–1^ beef extract, 10 g L^–1^ peptone, 20 g L^–1^ glucose, 2 g L^–1^ K_2_HPO_4_, 5 g L^–1^ anhydrous sodium acetate, 2 g L^–1^ ammonium citrate, 0.58 g L^–1^ MgSO_4_⋅7H_2_O, 0.25 g L^–1^ MnSO_4_⋅H_2_O, and 1 mL L^–1^ Tween 80. In this work, several modifications were made based on the traditional MRS: (1) Removal of polysaccharide-analogs in the three main components of the medium (beef extract, yeast extract, and peptone) before the preparation of the new medium. In brief, beef extract, yeast extract, and peptone were dissolved in pure water (10%, w/v), a quadruple volume of pre-chilled anhydrous ethanol was then added, and allowed to stand for 12 h at 4°C. After centrifugation (10,000 rpm, 15 min), the supernatant was concentrated and used for the preparation of the medium. (2) Replacement of glucose by other sugars as a carbon source by comparison (see Section “Statistical Optimization of Medium Composition and Culture Conditions for Exopolysaccharide Production”). (3) Addition of yeast nitrogen base as nitrogen source. (4) Optimization of dosage of nitrogen and carbon sources (see Section “Statistical Optimization of Medium Composition and Culture Conditions for Exopolysaccharide Production”).

### Statistical Optimization of Medium Composition and Culture Conditions for Exopolysaccharide Production

#### Single Factor Tests

To optimize the culture conditions for the EPS production by *L. rhamnosus* ZFM216, single factor tests were first performed to investigate the effects of culture time (0–32 h), culture temperature (27, 32, 37, 42, and 47°C), seed size (1, 3, 5, 7, and 9%), initial pH (5, 5.5, 6, 6.5, 7, 7.5, and 8), carbon source (glucose, fructose, galactose, maltose, sucrose, and lactose), and the dosage of nitrogen source (beef extract, yeast extract, peptone, and YNB) on the EPS yield.

#### Plackett–Burman Design

The Plackett–Burman experimental design, an economical and effective two-level experiment design method, can effectively screen out the main factors affecting the yield of EPS. According to the results of single factor tests (nine factors) maltose, beef extract, yeast extract, peptone, YNB, time, temperature, pH, and inoculum size were chosen to conduct Plackett–Burman design. Each factor was defined at two levels, a high level being denoted by (1) and a low level being denoted by (−1) (Table 1). The results of Plackett–Burman design were fitted by the first-order model as follows [Eq. (1)]:


(1) $$Y=\beta_{0}+\sum_{i=1}^{k}\beta_{i}\,X_{i}$$

**TABLE 1:** Plackett–Burman design and the results of EPS yield.

Run	Maltose	BE	YE	Pe	YNB	Time	Temp	pH	Seed	EPS yield
	
	*X*_1_	*X*_2_	*X*_3_	*X*_4_	*X*_5_	*X*_6_	*X*_7_	*X*_8_	*X*_9_	*Y*
1	−1	−1	−1	−1	−1	−1	−1	−1	−1	349.09
2	1	1	−1	−1	−1	−1	1	1	1	410.01
3	1	1	−1	1	−1	1	1	−1	−1	435.00
4	−1	1	1	−1	1	−1	1	−1	1	462.56
5	−1	1	−1	1	1	1	−1	1	1	371.87
6	−1	−1	1	1	−1	1	1	−1	1	356.32
7	1	1	1	−1	1	1	−1	−1	−1	487.91
8	1	−1	1	1	1	−1	1	1	−1	444.44
9	1	−1	−1	1	1	−1	−1	−1	1	432.69
10	−1	−1	−1	−1	1	1	1	1	−1	390.07
11	1	−1	1	−1	−1	1	−1	1	1	371.68
12	−1	1	1	1	−1	−1	−1	1	−1	351.87
High level	1 = 20	1 = 15	1 = 10	1 = 15	1 = 5	1 = 24	1 = 42	1 = 8	1 = 4	
Low level	−1 = 15	−1 = 12.5	−1 = 7.5	−1 = 10	−1 = 3	−1 = 20	−1 = 37	−1 = 7	−1 = 2	
Unit	g L^–1^	g L^–1^	g L^–1^	g L^–1^	g L^–1^	h	°C	/	%	(mg L^–1^)
*p*-value	0.0034 **	0.0100 *	0.0394 *	0.0461 *	0.0031 **	0.1649	0.0170 *	0.0091 **	0.0942	

*Means significance at the p < 0.05 level and **means significance at the p < 0.01 level.

where *Y* is the predicted yield of EPS, β*_0_* is the model intercept, β*_*i*_* is the linear coefficients, *X*_*i*_ is the independent variable, and *k* is the number of the factors. Nine assigned variables were screened in 12 runs; all experiments were carried out in triplicate and the average EPS yield was taken as a response. Design-Expert software (Version 8.0.6, Stat-Ease Inc., Minneapolis, MN, United States) was first used for the first-order model polynomial coefficient calculation and analysis, and then the significant variables were selected for further statistical analysis.

#### Box–Behnken Design

Box–Behnken design was conducted to further optimize the culture pH, the amount of maltose, and YNB for the EPS production. The design with 17 runs and the corresponding results are presented in Table 2, and the results can be explained by Eq. (2).


(2) $$Y=\beta_{0}+\sum_{i=1}^{k}\beta_{i}\,X_{i}+\sum_{i=1}^{k}\beta_{i\,i}\,X_{i}^{2}+\sum_{i=1}^{k}\beta_{i\,j}\,X_{i}\,X_{j}$$

**TABLE 2:** Box–Behnken design and the results of EPS yield.

N_*O*_.	Maltose	YNB	pH	EPS yield
	
	*X_1_/A*	*X*_5_/*B*	*X_8_/C*	*Y*(mg/L)
1	−1	0	−1	380.45
2	0	0	0	498.95
3	0	−1	1	421.12
4	1	1	0	445.12
5	−1	−1	0	353.24
6	0	−1	−1	353.11
7	−1	1	0	435.12
8	−1	0	1	390.16
9	0	1	−1	450.56
10	0	1	1	422.56
11	0	0	0	497.33
12	0	0	0	496.59
13	1	0	−1	432.56
14	0	0	0	486.12
15	1	0	1	432.52
16	1	−1	0	441.89
17	0	0	0	492.59
High level	1 = 25	1 = 7	1 = 7	
Low level	−1 = 15	−1 = 3	−1 = 6	
Unit	(g L^–1^L)	(g L^–1^)		(mg L^–1^)

where *Y* is the predicted EPS yield, β*_0_* is the model constant; β*_*i*_*, β*_*ii*_*, and β*_*ij*_* are linear, quadratic, and interaction coefficients, respectively; *X*_*i*_ and *X*_*j*_ are the independent factors; and *k* is the number of the factors. The second-order polynomial coefficient calculation and analysis were performed using Design-Expert software.

### Production of Exopolysaccharide

The fermentation of *L. rhamnosus* ZFM216 was performed using R-MRS (medium prepared with the pretreated materials), S-MRS (R-MRS containing 3 g L^–1^ of YNB), and M-MRS (the modified MRS medium under the optimum conditions), respectively. The method for the preparation of EPS was the same as that described in Section “Monitoring the Change in Polysaccharide Content in the Culture Medium During Fermentation.”

### Characterization of Exopolysaccharide

#### Chemical Composition

The content of total sugar of EPS from *L. rhamnosus* ZFM216 was determined by the phenol sulfuric acid method (16), protein was measured using Coomassie brilliant blue method (17), and sulfate was measured by barium sulfate turbidimetric method (18).

#### Monosaccharide Composition

The monosaccharide composition of EPS was determined by High-Performance Liquid Chromatography (HPLC) according to a published method with minor modifications (19). EPS samples (10 mg) were hydrolyzed with trifluoroacetic acid (5 mL, 2M). The hydrolyzate was derivatized with 1-phenyl-3-methyl-5-pyrazolone (PMP). HPLC system (Agilent 1100, Agilent, United States) was equipped with an Eclipse Plus C18 column (250 mm × 4.6 mm, 5 μm, Agilent, United States) and the detector wavelength was set at 250 nm. The mobile phase consisted of phosphate buffer (PBS, 0.09M PBS, pH 7.8) acetonitrile (83:17, v:v) at a flow rate of 1.0 mL min^–1^.

#### Molecular Weight Determination

The molecular weight of EPS was analyzed by gel permeation chromatography (GPC) (20). EPS sample (15 mg) dissolved in 1 mL ultrapure water was filtered through a 0.22-μm filter and put into a 5-mL sample bottle, and then ultrapure water containing NaNO_3_ (0.2 M) and NaH_2_PO_4_ (0.01 M) was added to the scale line and ultrasonicated. The ultrasonic dispersion was used for molecular weight determination. GPC system (Waters 1525 Isocratic HPLC Pump) was equipped with a PL aquagel-OH MIXED 8 μm chromatographic column and a differential detector (Waters 2414 Refractive Index Detector). Ultrapure water containing NaNO_3_ (0.2 M) and NaH_2_PO_4_ (0.01 M) was used as the mobile phase, the flow rate was 1 mL min^–1^, and the column temperature was 30°C. The standard sample was narrow distribution polyethylene glycol provided by Polymer Standards Service-USA, Inc., with molecular weights (Mp) of 330,000, 176,000, 82,500, 44,000, 25,300, 20,600, 12,600, 7130, 4290, 1400, 633, and 430, respectively, and the data were analyzed using Agilent GPC software.

#### Ultraviolet and Fourier Transform Infrared Spectroscopy Analysis

Ultraviolet spectra of EPS sample (1 mg mL^–1^) were obtained by recording on a microplate reader (Spectramax iD3, Molecular Devices, United States) in the range of 200–800 nm (21).

FT-IR spectrum of EPS sample was recorded on a Nicolet Is50 (Thermo Fisher, United States) using a KBr pellet method scanning in the range of 400 to 4000 cm^–1^ (21).

### Immunomodulatory Activity Assays

#### Cell Culture

Murine monocyte-macrophage RAW264.7 cells were incubated in a DMEM medium, supplemented with 1% penicillin-streptomycin and 10% fetal bovine serum, at 37°C in an incubator (MCO-175, Sanyo, Japan) containing 5% CO_2_.

#### Cell Viability Assay

Cell viability of RAW264.7 was investigated using the MTT assay (19). RAW264.7 cells (100 μL, 3 × 10^4^ cell/well) were seeded on a 96-well microplate and incubated at 37°C for 24 h. The supernatant was removed and the cells were washed gently with 200 μL of PBS twice. Then, the cells were treated with different concentrations of EPS solution (100 μL; 100, 200, 300, 400, 500, 600, and 700 μg mL^–1^ in DMEM) for 24 h. MTT (10 μL) was added and incubated for another 4 h, and the medium was then removed. DMSO (100 μL) was added to dissolve the insoluble formazan, and the absorbance of the resulting solution was measured at 570 nm. DMEM containing LPS (1 μg mL^–1^) served as a positive control, and DMEM without EPS served as a control. All assays were performed in quintuplicate.

#### Phagocytosis Assay

In this study, uptake of neutral red was used to assess the effect of EPS on the phagocytic activity of RAW 264.7 cells using a published protocol with minor modifications (22). RAW264.7 cells (100 μL, 3 × 10^5^ cell mL^–1^) were treated with different concentrations of EPS solution (100 μL; 100, 200, 300, 400, 500, 600, and 700 μg mL^–1^ in DMEM) in a 96-well microplate and incubated at 37°C in a 5% CO_2_ atmosphere for 24 h. LPS (1 μg mL^–1^) was used as a positive control. After removal of the supernatant, the cells were washed gently with PBS twice, and neutral red (100 μL, 0.1%, w/w) was then added to each well and continued to incubate for 1 h. Subsequently, the neutral red was removed and the cells were washed with PBS twice, followed by the addition of lysis buffer (200 μL, glacial acetic acid:ethanol = 1:1). After standing at room temperature for 2 h, the optical density was recorded at 540 nm.

#### Nitric Oxide and Cytokine Assays

RAW264.7 cells (1 mL, 3 × 10^5^ cell mL^–1^) were treated with different concentrations of EPS solution (1 mL; 100, 200, 300, 400, 500, 600, and 700 μg mL^–1^ in DMEM) in a 24-well microplate and incubated at 37°C for 24 h. LPS (1 μg mL^–1^) was used as a positive control. The concentrations of NO, IL-1β, IL-6, and TNF-α in the supernatant of the RAW264.7 cells were measured using a commercial NO assay kit and the corresponding ELISA kits according to the manufacturers’ instructions.

### Immunomodulatory Mechanism Analysis

#### Real-Time Quantitative PCR Assay

The mRNA expression of inducible nitric oxide synthase (iNOS) and cytokines were assessed by RT-qPCR (23). RAW264.7 cells (1 mL, 3 × 10^5^ cell mL^–1^) were treated with different concentrations of EPS solution (1 mL; 100, 300, and 500 μg mL^–1^ in DMEM) or LPS (1 μg mL^–1^) in a 24-well microplate and incubated at 37°C for 24 h. The cells were then washed with cold PBS twice and used for RNA extraction. The total RNA from the cells was extracted using commercial E.Z.N.A.^®^ Total RNA Kit II according to the manufacturers’ instructions. The quality and quantity of RNA were assessed by measuring the A260/A280 absorbance ratio using a NanoDrop 2000 spectrophotometer (Thermo Fisher Scientific, United States). RNA was reversed into cDAN using Hifari^®^ III 1st Strand cDNA Synthesis SuperMix (11141ES60, Yeasen, Shanghai, PRC) according to the manufacturer’s instructions. Then, the cDNA encoding iNOS, TNF-α, IF-1β, and IF-6 genes were analyzed by RT-qPCR using Hieff^®^ UNICON Blue qPCR SYBR Green Master Mix (11184ES08, Yeasen, Shanghai, China) according to the manufacturer’s instructions. The relative gene expression was calculated by the 2^–ΔΔCt^ method, and the housekeeping gene β-actin gene was used as an internal control. Primer sequences for the genes were listed in Supplementary Table 1.

#### Western Blot Analysis

Western blot analysis was performed according to a published method with minor modifications (24). In brief, RAW264.7 cells (2 mL, 3 × 10^5^ cell mL^–1^) were treated with different concentrations of EPS solution (1 mL; 100, 300, and 500 μg mL^–1^ in DMEM) or LPS (1 μg mL^–1^) in a six-well microplate and incubated at 37°C for 24 h, thereafter the total protein was extracted with 100 μL of lysis buffer (PMSF protease inhibitor: phosphatase inhibitor: RIPA lysis and extraction buffer = 1:1:100), the protein concentration was determined by BCA protein quantitative kit according to the manufacturers’ instructions, and stored at −20°C for standby. After denaturation in a boiling water bath, the proteins were separated by SDS-PAGE gel electrophoresis and transferred to PVDF membranes. The PVDF membranes were blocked with 5% BSA for 2 h, and then incubated with the corresponding primary antibodies against p65 (1:1000), p-p65 (1:1000), and GAPDH (1:500) at 4°C for 12 h, then incubated with the secondary antibodies IgG (1:8000) for 1 h at 37°C. The bands were visualized with a FluorChem HD2 system (ProteinSimple, United States) after treatment with ECL luminous Kit. ImageJ software was used to determine the gray value for quantitative analysis.

#### Immunofluorescence Assay

The immunofluorescence staining analysis of nuclear translocation of NF-κB was performed according to the literature with minor modifications (25). RAW264.7 cells (2 mL, 3 × 10^5^ cell mL^–1^) were treated with EPS solution (1 mL; 500 μg mL^–1^ in DMEM) or LPS (1 μg mL^–1^) on a laser confocal dish and incubated at 37°C for 24 h. The cells were then fixed with 4% paraformaldehyde for 12 h, further treated with 0.5% Triton X-100 for 20 min and blocked with 5% BSA at room temperature for 1 h. The cells were incubated with the p65 primary antibody (1:100) at 4°C for 12 h and then incubated with the Anti-rabbit IgG (H + L) F(ab’)2 Fragment (Alexa Fluor 488 Conjugate) at 37°C for 1 h in the dark. The nuclei were stained with DAPI for 5 min, and the cells were observed and photographed with a laser confocal microscope (Olympus, Japan).

### Statistical Analysis

One-way analysis of variance (ANOVA) was performed using SPSS version 25 (SPSS Inc., Chicago, IL, United States) to compare the results. Duncan’s multiple range test was used for analyzing the significance of the differences. If *p* < 0.01, the differences were considered statistically significant. All the data were expressed as mean ± standard deviation (SD).

## Results and Discussion

### Effect of MRS Medium on Bacterial Growth and Exopolysaccharide Production

**FIGURE 1:**
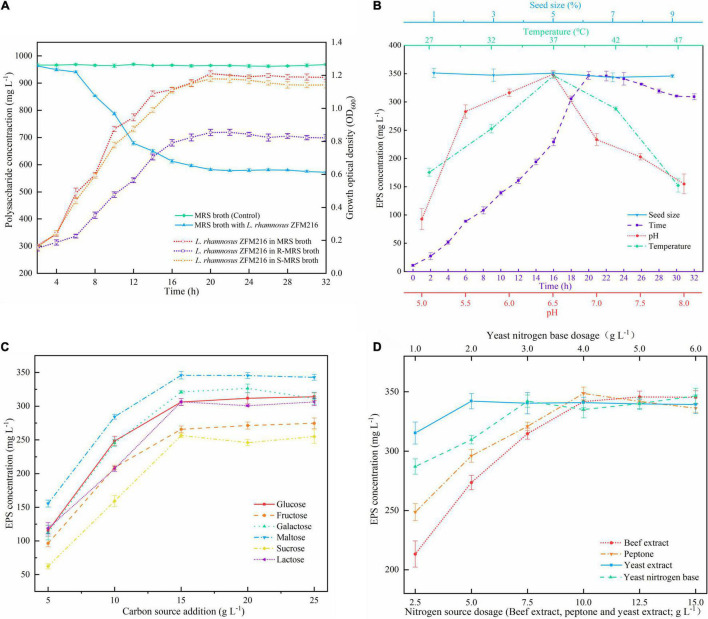
**(A)** Change in polysaccharide content in MRS broth and growth curves of the *Lacticaseibacillus rhamnosus* ZFM216 strain in different media. R-MRS means the polysaccharide-analogs-removed MRS medium, S-MRS means R-MRS supplied with 3 g L^– 1^ of YNB. **(B)** Effects of seed size, time, pH, and temperature on the EPS production in the single factor tests. **(C)** Effect of carbon sources and their dosage on EPS production. **(D)** Effect of nitrogen source dosage on EPS production.

As presented in Figure 1A, the polysaccharide content in MRS broth in the presence of *L. rhamnosus* ZFM216 decreased over time, and the final polysaccharide content was 582.30 ± 4.85 mg L^–1^, while the polysaccharide content in MRS broth without any strain remained almost unchanged (964.49 ± 3.96 mg L^–1^). The result indicated that some polysaccharide-analogs were metabolized by the strain. The polysaccharides presented in MRS broth with *L. rhamnosus* ZFM216 included the EPS produced by the strain and polysaccharide-analogs derived from the medium. Vaningelgem et al. (26) found that the glucomannans presented in yeast extract and peptone interfered with the quantification of the EPS produced during the fermentation. Some low molecular weight sugars can be removed by dialysis, but most polysaccharide-analogs cannot be separated from the EPS. It was found that beef extract, yeast extract, and peptone in 1 L of MRS broth contained 489.38 ± 7.83, 312.25 ± 5.12, and 134.02 ± 5.34 mg of polysaccharide analogs, respectively, accounting for about 97% of the total polysaccharide in MRS broth. Therefore, it is essential to remove the polysaccharide analogs before preparing the new medium for fermenting the strain to produce EPS.

It was found that the growth state of the bacteria, cultured with the polysaccharide-removed MRS medium (R-MRS), was lower than that in the traditional MRS broth (Figure 1A), and the yield of EPS was only 281.07 ± 5.90 mg L^–1^. This is possible because some necessary components for bacterial growth were also removed when removing the polysaccharides from beef extract, yeast extract, and peptone. When S-MRS (R-MRS supplied with 3 g L^–1^ of YNB) was used for bacterial culture, the growth state of the bacteria was comparable to that of the traditional MRS medium (Figure 1A), and the yield of EPS was 340.14 ± 6.54 mg L^–1^. On the basis of the result, statistical optimization was further used to obtain the optimal formulation of the medium and culture conditions for the EPS production by *L. rhamnosus* ZFM216.

### Statistical Optimization for Exopolysaccharide Production

#### Single Factor Tests

The effects of temperature, time, initial pH, seed size, carbon source, and the dosage of nitrogen source on the yield of EPS were investigated by the single factor tests. As illustrated in Figures 1B–D, optimal conditions for EPS production were as follows: incubation temperature 37°C, culture time 20 h, pH 6.5, maltose (15 g L^–1^) as carbon source, and BE (12.5 g L^–1^), YE (7.5 g L^–1^), Pe (10 g L^–1^), and YNB (3 g L^–1^) as nitrogen source. The seed size was found to have no obvious effect on the yield of EPS, thus 1% of the seed size was used in the following investigation.

#### Plackett–Burman Design

A Plackett–Burman design analysis was performed for each factor at two levers for 12 trials, and the corresponding EPS production are presented in Table 1. Based on the Design Expert analysis, a first-order model expressing the EPS yield was obtained as follows [Eq. (3)]:


(3) $$Y=373.37+25\,X_{1}+29.16\,X_{2}+8.61\,X_{3}-6.59\,X_{4}+26.3\,X_{5}\,-\,3.15\,X_{6}+11.11\,X_{7}-15.3\,X_{8}-4.44\,X_{9}$$

where X_1_, X_2_, X_3_, X_4_, X_5_, X_6_, X_7_, X_8_, and X_9_ represent dosages of maltose, BE, YE, Pe, and YNB, time, temperature, initial pH, and seed size, respectively.

Statistical analysis of the EPS production showed that the *p*-value (<0.01) was low, implying the extreme significance of the model. The high determinant coefficient *R*^2^ of the model (0.9979) indicated that the model simulated the test process well. The influences of maltose concentration (*X*_1_), YNB (*X*_5_), and pH (*X*_8_) were extremely significant (*p* < 0.01) on EPS production, while the effects of BE (*X*_2_), YE (*X*_3_), Pe (*X*_4_), and temperature (*X*_7_) were significant (*p* < 0.05).

#### Box–Behnken Design

According to the results of the Plackett–Burman design, the three factors which had an extremely significant effect on the EPS yield, namely maltose (*A*), YNB (*B*), and pH (*C*) were selected for further optimization by Box–Behnken design. The results and the ANOVA of the models are presented in Table 3. The *F*-value of the model was high (115.02) and the *p-*value was extremely low (<0.0001), suggesting that the model was adequate. The value of the regression coefficient *R*^2^ was 0.9933, implying that the model fitted the test process well, while *R*^2^_*adj*_ (0.9846) explained more than 98% of the reliability in EPS production. The *p*-value for lack of fit (0.2908) indicated the insignificance of lack of fit compared with the pure error. All the results showed a good consistency with the predicted yield of EPS, suggesting the validity of the mathematical model. The linear coefficients (*A*, *B*, and *C*), the interaction term coefficients (*AB* and *BC*), and the coefficients of the quadratic term (*A*^2^, *B*^2^, and *C*^2^) had a significant effect on the yield of EPS (*p* < 0.01).

**TABLE 3:** ANOVA for the EPS production according to response surface quadratic model.

Source	Sum of squares	df	Mean square	*F*-value	*p*-value
Model	36507.46138	9	4056.384597	115.0200761	<0.0001
*A*	4661.9168	1	4661.9168	132.1901344	<0.0001
*B*	4232	1	4232	119.9997067	<0.0001
*C*	308.5128	1	308.5128	8.74797862	0.0212
*AB*	1546.455625	1	1546.455625	43.85024137	0.0003
*AC*	23.765625	1	23.765625	0.67388186	0.4388
*BC*	2304.480025	1	2304.480025	65.34426445	<0.0001
*A* ^2^	6468.165192	1	6468.165192	183.4068823	<0.0001
*B* ^2^	5541.827287	1	5541.827287	157.1402763	<0.0001
*C* ^2^	8986.824002	1	8986.824002	254.8242544	<0.0001
Residual	246.86727	7	35.26675286		
Lack of fit	140.98455	3	46.99485	1.775354845	0.2908
Pure error	105.88272	4	26.47068		
Cor total	36754.32865	16			
*R*^2^ = 0.9933	*R*^2^_*adj*_ = 0.9846	Pred *R*^2^ = 0.9341	C.V. (%) = 1.87		

A second-order polynomial equation model by multiple regression analysis was obtained by fitting the experimental data as Eq. (4):


(4) $$Y=494.32+24.14\,A+23\,B+6.21\,C-19.66\,A\,B-2.44\,A\,C\,-\,24\,B\,C-39.19\,A^{2}-36.28\,B^{2}-46.2\,C^{2}$$

**FIGURE 2:**
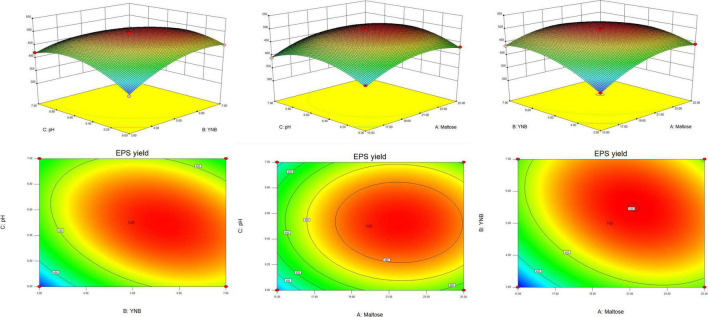
The response surface and corresponding contour plots show the effects of variables on the response (yield of EPS).

The optimal level of the three variables and their interactions were visualized by the three-dimensional response surface methodology and the count plots in the function of two variables (Figure 2). The elliptical shape of the contour plots indicated that the interactions between the variables were significant. The optimal value of the variables calculated by the equation model was as follows: maltose 21.23 g L^–1^, YNB 5.51 g L^–1^, and pH 6.5, and the predicted maximum production of EPS reached 500.16 g L^–1^. Comprehensively, the optimized composition of the M-MRS was as follows: 7.5 g L^–1^ yeast extract, 12.5 g L^–1^ beef extract, 10 g L^–1^ peptone, 21.23 g L^–1^ maltose, 5.51 g L^–1^ YNB, 2 g L^–1^ K_2_HPO_4_, 5 g L^–1^ anhydrous sodium acetate, 2 g L^–1^ ammonium citrate, 0.58 g L^–1^ MgSO_4_⋅7H_2_O, 0.25 g L^–1^ MnSO_4_⋅H_2_O, 1 mL L^–1^ Tween 80, and initial pH was 6.5. The optimized culture conditions for the production of EPS by *L. rhamnosus* ZFM216 were ascertained as follows: seed size 1%, culture temperature 37°C, and time 20 h. Under these conditions, the actual EPS yield was 496.64 ± 3.15 mg L^–1^, which was close to the predicted value, indicating that the model could be used to optimize the production of EPS from the strain. Furthermore, the EPS yield under the optimized condition was 76.70% higher than that in the unoptimized conditions (281.07 ± 5.90 mg L^–1^). The improvement of fermentation strategies helps to enhance EPS yield. Therefore, it is essential to improve or develop novel fermentation techniques for microbial EPS production. Moreover, the use of statistical models in fermentation helps to obtain the maximum EPS yields (27).

### Characterization of Exopolysaccharide

#### Chemical Composition, Monosaccharide Composition, and Molecule Weight

**FIGURE 3:**
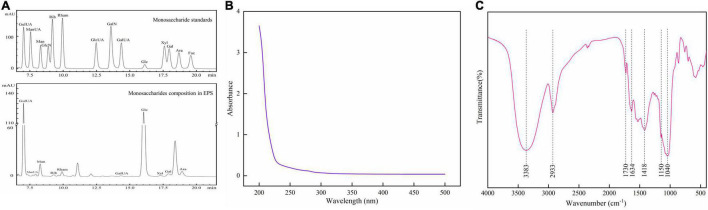
Monosaccharide composition analysis by HPLC **(A)**, UV spectrum **(B)**, and FT-IR spectrum **(C)** of EPS.

The contents of total sugar, protein, and sulfate were determined to be 70.06 ± 4.21%, 1.44 ± 0.11%, and 17.74 ± 1.02%, respectively. As shown in Figure 3A, EPS was composed of guluronic acid, mannuronic acid, mannose, ribose, rhamnose, galacturonic acid, glucose, xylose, galactose, and arabinose with the molar ratios of 95.2:1:19.2:1.4:3.3:1.2:106.8:1.4:5.9:13.6. Glucose and guluronic acid residues were found to be predominant in EPS.

The GPC analysis indicated that EPS had a weight average molecular weight (Mw) of 32.2 kDa and a number average molecular weight (Mn) of 19.9 kDa, with a polydispersity index (PDI) of 1.61. This result agrees with some previous studies, in which the Mw of EPS from *L*. *rhamnosus* was reported to be 10^4^ to 10^5^ Da (28, 29), but differs from that of Calsteren’s research (10^7^ Da) (30).

#### Ultraviolet and IR Analysis

No obvious absorptions at 260, 280, and >400 nm in the UV spectrum of EPS (Figure 3B) indicated that EPS contained almost no nucleic acid, proteins (<3%), and pigments, suggesting the high purity of EPS.

In the FT-IR spectra of EPS (Figure 3C), a strong absorption peak appearing at 3,383 cm^–1^ is ascribed to the hydroxyl group (31); the peak at 2,933 cm^–1^ is caused by an asymmetrical C-H stretching vibration (32); and the peaks at 1,634 and 1,418 cm^–1^ represent the asymmetric and symmetric stretching of COO- group (33), confirming the existence of uronic acids. Meanwhile, the presence of the signal at 1,730 cm^–1^ indicates the testification of some uronic acid residues in EPS (34). Two successive peaks around 1,150 and 1,040 cm^–1^ are the characteristics of C-O vibration (C-O-H, C-O-C) in the pyranose ring (35).

### Immunomodulatory Activity of Exopolysaccharide

#### Effect of Exopolysaccharide on the Viability of RAW264.7 Cells

**FIGURE 4:**
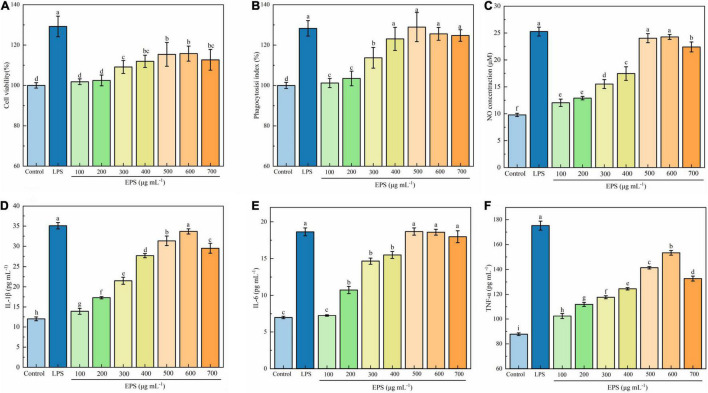
Effect of EPS on cell viability **(A)**, phagocytosis **(B)**, NO production **(C)**, IL-1β **(D)**, IL-6 **(E)**, and TNF-α **(F)** secretion of RAW 264.7 cells. The data are expressed as mean ± SD, different lower-case letters indicate the significant difference between the results at different EPS concentrations (*p* < 0.01).

Macrophages are a major class of innate immune cells in the inflammatory response and play an important role in innate and adaptive immune responses. When the body is stimulated by inflammatory injury, there will be an elevated proliferation of macrophages in the tissue, which promotes the immune response driven by the specific microenvironment (27, 36, 37). Generally, when the cell survival rate is less than 90%, the sample is considered to be cytotoxic (38). As presented in Figure 4A, EPS was able to promote the growth of RAW264.7 cells in a dose-dependent manner in the concentration range of 100–600 μg mL^–1^. Maximum viability was observed in the presence of 600 μg mL^–1^ EPS, being 115.76% of the control group, but it was lower than that of the positive control (129.22%). In the report of Li et al. (36) a polysaccharide isolated from the Antarctic bacterium *Pseudoalteromonas* sp. S-5 insignificantly affected the viability of RAW 264.7 cells over the entire concentration range of 2.5–500 μg mL^–1^. Polysaccharides from different sources may have different effects on the growth of macrophages. Li et al. (39) found that the degraded polysaccharide from *Porphyra haitanensis* had to promote effect on the cell proliferation within a certain concentration range; while an exopolysaccharide isolated from a coral-associated fungus inhibited the viability of RAW 264.7 cells when the concentration of polysaccharide exceeded 250 μg mL^–1^ (40).

#### Effect of Exopolysaccharide on the Phagocytic Activity

Phagocytosis is one of the defense mechanisms of organisms, which eliminates bacteria, viruses, and foreign particles in the tissue fluids and blood (41). Phagocytic activity has been widely used as an index to evaluate the immunoregulatory activity of macrophages. As shown in Figure 4B, the phagocytic activity of the macrophages increased with increasing the EPS concentration up to 500 μg mL^–1^, after which the effect decreased slightly. The maximum phagocytosis was observed at a concentration of 500 μg mL^–1^, being 128.92% of the control group, which was comparable to that of positive control LPS (*p* < 0.01). The finding in the present work is similar to that of Wang et al. (22), who reported that exopolysaccharides produced by *Lactobacillus plantarum* JLK0142 significantly promoted the phagocytic activity of RAW 264.7 cells, and exhibited the best effect at 500 μg mL^–1^. Polysaccharides from *Hericium erinaceus* fruiting bodies (42) and *Rhodopseudomonas palustris* (43) were also found to possess an enhancing effect on the phagocytic activity.

#### Effect of Exopolysaccharide on Nitric Oxide Production

Nitric oxide is an important substance involved in the antibacterial activity and antitumor immune response of macrophages (44). Activated macrophages can increase the activity of nitric oxide synthase, and the degree of immune response can be reflected by the NO concentration. As presented in Figure 4C, EPS significantly induced NO production of the RAW 264.7 cells (*p* < 0.01). The NO production reached a maximum at EPS concentrations of 500 and 600 μg mL^–1^, being 24.04 and 24.07 μM respectively, which was close to that in LPS-treated group (*p* > 0.05). Such an increase in NO production suggests that EPS may activate the tumoricidal and bactericidal activities of macrophages and play a vital role in the host defense system. Polysaccharides from *Elaeagnus angustifolia* L. pulp were found to be able to significantly increase the production of NO in the concentration of 25 to 400 μg mL^–1^(44). It has also been reported that polysaccharides from different sources, such as *Sargassum fusiforme, Porphyra yezoensis*, and *Brassica rapa* L., significantly increase the NO production of RAW 264.7 cells (45).

#### Effect of Exopolysaccharide on Production of Cytokines

TNF-α, IL-1β, and IL-6 are produced by the activated macrophages and play a vital role in immune responses (43). As proinflammatory cytokines, TNF-α, IL-1β, and IL-6 can protect the host from external infection and repair damaged tissues and cells.

As shown in Figures 4D–F, the contents of IL-1β, IL-6, and TNF-α in EPS-treated cells were significantly higher than those in the untreated macrophage group (control) (*p* < 0.01). Both contents of TNF-α and IL-1β significantly increased with the increase of EPS concentration up to 600 μg mL^–1^ (*p* < 0.01), thereafter the values decreased (*p* < 0.01); while the content of IL-6 significantly increased with the increase of EPS concentration up to 500 μg mL^–1^ (*p* < 0.01), thereafter the value remained unchanged. The maximum content of TNF-α observed in the EPS group (153.30 pg mL^–1^) was still lower than that of positive control LPS (175.22 pg mL^–1^). The maximum contents of IL-1β and IL-6 of the EPS group (33.67 and 18.67 pg mL^–1^, respectively) were close to the corresponding values of LPS (35.09 and 18.64 pg mL^–1^, respectively). It can be concluded that EPS can stimulate RAW 264.7 cells and promote their secretion of cytokines, thereby exerting immune-enhancing effect. Zhou et al. (46) also found that polysaccharides from *Shiraia bambusicola* increased the production of IL-1β, IL-6, and TNF-α in a dose-dependent manner at 50–200 μg mL^–1^. EPS from *L. rhamnosus* RW-9595 M could induce immunosuppression by increasing the production of pro-inflammatory cytokines, including TNF-α and IL-6 (47).

### Immunomodulatory Mechanism

#### Effect of Exopolysaccharide on mRNA Expression of Inducible Nitric Oxide Synthase and Cytokines

**FIGURE 5:**
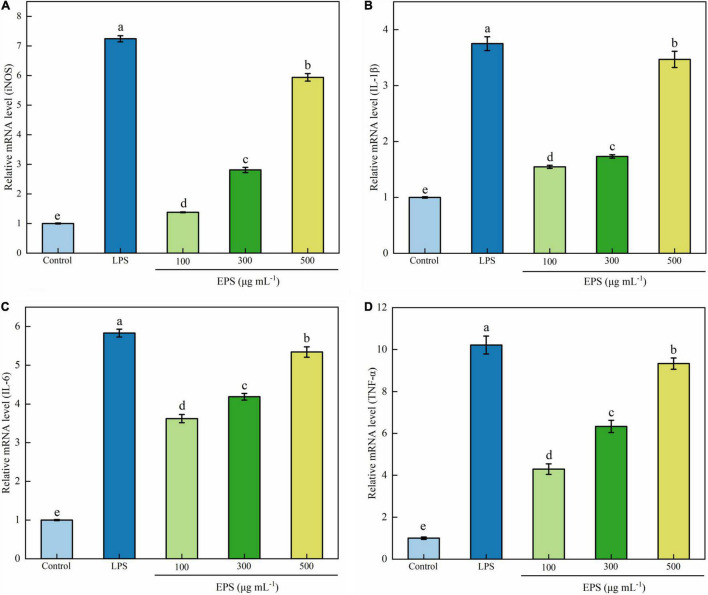
Effects of EPS on mRNA expression of iNOS **(A)**, IL-1β **(B)**, IL-6 **(C)**, and TNF-α **(D)** in RAW264.7 cells. The data are expressed as mean ± SD, different lower-case letters indicate the significant difference between the results at different EPS concentrations (*p* < 0.01).

The activation of macrophages and the enhancement of immune activity are closely related to the expression of genes encoding immune-related cytokines. To investigate the immune-regulating mechanism of EPS at the molecular level, the effects of EPS on mRNA expression of inducible NO synthase (iNOS), TNF-α, IL-1β, and IL-6 were quantitatively analyzed using RT-qPCR. As shown in Figures 5A–D, EPS significantly increased the mRNA expression of iNOS and three cytokines at the test concentrations (100–500 μg mL^–1^). Immunomodulatory EPS upregulated mRNA expression of related enzymes (27). NO is an important signal molecule of immune response; thus, it is likely that EPS promotes immunomodulatory activity by increasing mRNA expression of iNOS (48). Zhang et al. (43) also found a polysaccharide from *R. palustris* named REEPSs-30 which increased the mRNA expression of TNF-α, IL-1β, and IL-6, and the effect was also positively correlated with the concentration of polysaccharide. A novel polysaccharide from *H. erinaceus* fruiting bodies could significantly stimulate the cell secretion of NO, IL-6, and TNF-α in a dose-dependent manner (at 100–1,000 μg mL^–1^) (42).

#### NF-κB Signaling Pathway

Nuclear factor kappa-B (NF-κB) is a family of proteins composed of complex polypeptide subunits. It usually forms a homologous or heterodimer from p65 and p50 and is inactivated in the cytoplasm due to the combination with the inhibitory protein complex IκB-α (49). The NF-κB might be activated through two major pathways, a canonical and non-canonical (alternative) pathway. EPS has been demonstrated to stimulate macrophages via activation of NF-κB through a canonical signaling pathway through degradation of IκB-α (27). After activation, NF-κB enters the nucleus and participates in protein transcription and translation. Thus, it is closely related to immune response, tumorigenesis, and apoptosis regulation (50, 51). The NF-κB signaling pathway is also involved in the stimulation of mRNA expression of iNOS, IL-1β IL-6, TNF-α, and the secretion of NO and cytokines during an immune response (52). The activation of NF-κB can be determined by detecting the protein phosphorylation level of the main subunit p65 by Western blot and transportation of p65 into the nucleus by immunofluorescence.

**FIGURE 6:**
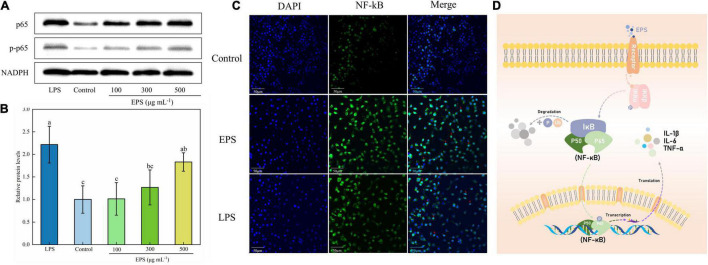
Effect of NF-κB signaling pathway on EPS-induced macrophage activation. **(A)** The effect of EPS on the level of phosphorylation of p65 in RAW264.7 cells by Western blotting. **(B)** Relative quantitative analysis of p-p65/p65 protein phosphorylation levels. **(C)** Effect of EPS on p65 nuclear status in RAW264.7 cells. **(D)** Schematic diagram of immune regulation mechanism of EPS by NF-κB signaling pathway. The data are expressed as mean ± SD, different lower-case letters indicate the significant difference between the results at different EPS concentrations (*p* < 0.01).

Western blot analysis showed that the ratio of key proteins phosphorylated p65 (p-p65) to p65 was significantly increased compared to the blank group (Figures 6A,B), suggesting the activation of the NF-κB signaling pathway in RAW264.7 cells. Moreover, the degree of activation was positively correlated with the concentration of EPS. Similarly, in the research of Zhu et al. (52), the phosphorylation ratio of p65 was significantly increased when treated with the polysaccharide LGP-1 from Guapian tea, showing an increasing trend with the increase of concentration.

In the immunofluorescence imaging (Figure 6C), the blue region represented the nuclear staining area, and the green region was the p65 protein fluorescence. The p65 green fluorescence of the blank group was weak, and after EPS treatment, the green fluorescence was significantly enhanced and the merged image showed that a large number of purple co-localization regions were represented in the nucleus. Similar to the result of Li et al. (36), who used red fluorescence to label the protein p65, the merged image showed a small amount of p65 subunit in the nucleus of the control group. The red fluorescence of protein p65, with bright and concentrated distribution, can be found in the groups treated with LPS and polysaccharides (200 μg mL^–1^). In conclusion, EPS could activate macrophages through the NF-κB pathway (Figure 6D), resulting in an immune response and increased release of cytokines.

## Conclusion

A novel culture medium for EPS-producing *L. rhamnosus* ZFM216 was successfully developed. Using this medium, the interference of polysaccharides derived from the conventional culture medium can be avoided in the quantification of EPS produced during fermentation. Under the optimized culture conditions, the EPS yield reached 496.64 ± 3.15 mg L^–1^, which was 76.70% higher than that obtained under unoptimized conditions (281.07 ± 5.90 mg L^–1^). The EPS investigated in this work possessed good effects in enhancing the proliferation, phagocytosis, and the release of NO and cytokines of RAW264.7 macrophages, suggesting the good immunomodulatory activity of EPS. Upregulating the mRNA expression of iNOS, TNF-α, IL-1β, and IL-6 could be the causative immunomodulatory mechanism of EPS. EPS was also found to initiate and activate the immune signal transduction through the NF-κB signaling pathway. EPS could be a potential candidate for a functional food supplement for the hypoimmunity population.

## Data Availability Statement

The raw data supporting the conclusions of this article will be made available by the authors, without undue reservation, to any qualified researcher.

## Author Contributions

LC designed and performed the experiments, conducted the data analysis, and wrote—original draft of the manuscript. QG involved in the supervision of the study and resources. TZ contributed to conceptualization, supervision, funding acquisition, and writing—review and editing. All authors contributed to the article and approved the submitted version.

## Conflict of Interest

The authors declare that the research was conducted in the absence of any commercial or financial relationships that could be construed as a potential conflict of interest.

## Publisher’s Note

All claims expressed in this article are solely those of the authors and do not necessarily represent those of their affiliated organizations, or those of the publisher, the editors and the reviewers. Any product that may be evaluated in this article, or claim that may be made by its manufacturer, is not guaranteed or endorsed by the publisher.
