# Supplementary material for: Statistical Optimization of Novel Medium to Maximize the Yield of Exopolysaccharide From Lacticaseibacillus rhamnosus ZFM216 and Its Immunomodulatory Activity
Source: Front Nutr. 2022 Jun 2;9:924495. doi: 10.3389/fnut.2022.924495 (PMC9201479; doi:10.3389/fnut.2022.924495)
Supplement: Supplementary file 1 [file Table_1.DOCX]

Supplementary Material

# Supplementary Table 1S

**Table 1S.** The sequence of primers

| Name | | Nucleotide sequences of primer |
| --- | --- | --- |
| β-actin | Forward | 5'-TGGAATCCTGTGGCATCCATGAAAC-3' |
|  | Reverse | 5'-TAAAACGCAGCTCAGTAACAGTCCG-3’ |
| iNOS | Forward | 5'-CCCTTCCGAAGTTTCTGGCAGCAGC-3' |
|  | Reverse | 5'-GGCTGTCAGAGCCTCGTGGCTTTGG-3' |
| TNF-α | Forward | 5'-ATGAGCACAGAAAGCATGATC-3' |
|  | Reverse | 5'-TACAGG CTTGTCACTCGAATT-3’ |
| IL-1β | Forward | 5'-ATGGCAACTATTCCTGAACTCAACT-3' |
|  | Reverse | 5'-CAGGACAGGTATAGATTCTTTCCTTT-3’ |
| IL-6 | Forward | 5'-TTCCTCTCTGCAAGAGACT-3' |
|  | Reverse | 5'-TGTATCTCTCTGAAGGACT-3' |
